# Central Nervous System Involvement in Childhood Acute Lymphoblastic Leukemia: An Analysis of Day-One Versus Day-Eight Lumbar Punctures in Remission Induction Therapy

**DOI:** 10.7759/cureus.12464

**Published:** 2021-01-03

**Authors:** Uzma Arshad, Naeem Jabbar, Neelum Mansoor, Maryam Haider, Zainab Butt, Kishwer Nadeem

**Affiliations:** 1 Pediatrics, Jinnah Medical and Dental College, Karachi, PAK; 2 Department of Pediatric Oncology, The Indus Hospital, Karachi, PAK; 3 Department of Hematology, The Indus Hospital, Karachi, PAK; 4 Pediatrics, Dr. Ruth K. M. Pfau, Civil Hospital, Karachi, PAK

**Keywords:** acute lymphoblastic leukemia, remission induction therapy, leukemic blast cells, central nervous system, lumbar puncture, cerebrospinal fluid

## Abstract

Introduction

Traumatic lumbar puncture (TLP+) can lead to the iatrogenic infiltration of the central nervous system (CNS) by circulating leukemic blast cells in childhood acute lymphoblastic leukemia (ALL). The risk of TLP+ is increased by a number of factors at the time of presentation of the disease, such as a high white cell count (WCC), T-ALL phenotype, and unstable clinical condition of the patient. For this reason, the first lumbar puncture (LP) was deferred until Day Eight of prednisolone prophase during remission induction therapy in one set of patients. The objective was to compare the historical cohort of Day-One LP with Day-Eight LP with respect to the incidence of TLP+ and de novo CNS leukemia.

Methods

A retrospective comparative data analysis of 1,185 childhood ALL patients aged 1-16 years was conducted based on the electronic medical records of the pediatric hematology-oncology department of The Indus Hospital (TIH), Karachi, from January 2010 to August 2018. A total of 600 patients whose LP was done on Day One (January 2010-May 2015) were placed in cohort A, whereas 585 patients whose LP was performed on Day Eight (June 2015-August 2018) were placed in cohort B. After the examination of the cerebrospinal fluid (CSF), the status of CNS infiltration was classified as CNS-1, CNS-2, CNS-3, and TLP+.

Results

A total of 1,185 patients were included in the study, of whom 600 patients were in cohort A and 585 patients in cohort B. The incidence of TLP+ was found to be lower in cohort B (1.7%) as compared with the incidence in cohort A (4.3%) (p-value=0.009). However, there was an increase in the incidence of CNS-3 cases in cohort B (8%) as compared to cohort A (3%) (p-value: <0.001). When the CNS status of both the cohorts was compared with that of the internationally published data, a low incidence of TLP+ cases was noted in patients with LP on Day Eight.

Conclusion

The modified approach of performing the first LP on Day Eight significantly reduced the incidence of TLP+ cases. However, an unusual finding of a significant increase in the CNS-3 leukemia was noted. More prospective studies are needed to investigate this significant increase in CNS-3 cases.

## Introduction

The overall survival in childhood acute lymphoblastic leukemia (ALL) has considerably improved to 80-85% over the last few decades [1-3]. However, the relapse of the disease in the bone marrow and/or central nervous system (CNS) is still a major cause of treatment failure and accounts for 15-20% of the cases [4].

The infiltration of CNS by leukemic blast cells can be either de novo or iatrogenic. The de novo CNS infiltration occurs when blast cells metastasize the cerebrospinal fluid (CSF) as a result of the natural course of primary disease, whereas the iatrogenic infiltration occurs secondary to a traumatic lumbar puncture (TLP+), which results in the contamination of CSF with blast cells. CNS-3 is defined as the infiltration of CNS with pleocytosis (total CSF leukocyte count of ≥5/uL), whereas CNS-2 is defined as the infiltration without pleocytosis (total CSF leukocyte count of <5/uL). While CNS-3 is considered an independent marker of poor prognosis, CNS-2 has been reported to have a variable prognosis in various clinical trials [5-8]. The prognostic impact of TLP+ on the CNS infiltration was extensively studied in the past and has been shown to have adverse effects on the overall outcome of the disease secondary to CNS relapse [6,9].

More than 250 new cases of ALL are registered every year in the department of pediatric hematology-oncology of The Indus Hospital (TIH), Karachi, with the majority being classified as high risk according to the National Cancer Institute (NCI) criteria [10]. The published data shows that 34% of all the ALL cases presented with a total white cell count (WCC) of more than 50 x 10^9^ white blood cells/L [11]. The high WCC may have been caused by a delay in the recognition and referral of patients, and it leads to an unstable clinical condition among the patients. These adverse clinical factors at the time of presentation of disease along with limited supportive services are associated with an increased risk of traumatic taps when lumbar puncture (LP) is performed on Day One of remission induction therapy [11]. For this reason, in the present study, LP on Day One was deferred. Instead, prednisolone monotherapy was given during the first week of the remission induction phase followed by a diagnostic LP on Day Eight. The rationale was to clinically stabilize the patients by decreasing the number of circulating blast cells substantially before performing the first LP with the use of therapies like antibiotics, blood transfusions, and nutritional support, and thereby analyze the impact of this approach on the incidence of TLP+. The data was further compared with the historical cohorts of upfront (Day-One) LP [6,9] to ascertain the differences in the incidence of TLP+ and de novo CNS leukemia. The same approach was used by Hasegawa et al. in a randomized controlled trial where they delayed the first LP till Day Eight in 754 patients and observed a significant reduction in the incidence of TLP+ [12].

## Materials and methods

This retrospective comparative study was conducted at the pediatric hematology-oncology department of The Indus Hospital (TIH), Karachi. After obtaining approval from the hospital’s ethical review committee (IRB number: IRD_IRB_2019_05_002), data was reviewed through the electronic medical records. All children aged 1-16 years who were diagnosed with ALL from January 2010 to August 2018 were included in the study. The diagnosis of ALL in these patients was established through the examination of the peripheral blood film and/or bone marrow samples, by using the techniques of immunohistochemistry or flow cytometry. Children diagnosed with infantile leukemia were excluded from the study due to the differences in pathophysiology, cytogenetics, and treatment protocols. Those ALL patients who had their bone marrow examination done elsewhere were also excluded from the study.

A total of 1,158 patients were analyzed. These patients were further classified into two groups: cohort A and cohort B. Cohort A consisted of 600 patients who were diagnosed with ALL between January 2010 and May 2015, whereas cohort B comprised 585 patients diagnosed between June 2015 and August 2018. In cohort A, the first LP was done on Day One, followed by an upfront multi-agent chemotherapy according to the Children’s Oncology Group (COG) ALL treatment protocol. In cohort B, the Day-One LP was deferred. Instead, a monotherapy with prednisolone was given for one week with a dose of 60 mg/m^2^/day, followed by an LP on Day Eight. This treatment was based on the Berlin-Frankfurt-Münster (BFM) ALL protocol, with the modification of delaying the first LP until Day Eight of treatment. In both groups, the CSF samples obtained through LP were examined within two hours of collection for the total CSF leukocyte count and the presence of leukemic blast cells. Based on the results of CSF analysis, the status of CNS infiltration with blast cells was classified as CNS-1, CNS-2, CNS-3, and TLP+. CNS-1 is defined as the absence of blast cells; CNS-2 is the presence of blast cells with a total CSF leukocyte count of <5/uL; CNS-3 is the presence of blast cells with a total CSF leukocyte count of ≥5/uL, and TLP+ is the presence of blast cells with a total CSF leukocyte count of <5/uL, and ≥10 red blood cells/uL [9].

Data were entered into and analyzed using SPSS Statistics version 21.0 (IBM Corp., Armonk, NY). Descriptive statistics like mean and standard deviations were calculated for age and WCC. Frequencies and percentages were calculated for qualitative variables like CNS status (CNS-1, CNS-2, CNS-3, TLP+), the phenotype of the disease [T-cell ALL, B-cell ALL, ALL not otherwise specified (NOS)], and National Cancer Institute (NCI) risk (standard, high) [10]. Chi-squared test was applied to compare the results of current data (cohort B) to historical data (cohort A). The CNS status of cohort B was further compared to the risk variables like age, WCC, NCI risk, and phenotype. Both cohorts were compared with the internationally published data [6,9,11] to ascertain the differences in the incidence of TLP+ and de novo CNS leukemia. A p-value of less than 0.05 was considered statistically significant.

## Results

A total of 1,185 patients were included in the study, of whom 600 patients were in cohort A and 585 patients in Cohort B. The mean age of the patients in cohort A and B was 7.3 ± 4.1 and 7.2 ± 4.1 years, respectively. A comparison of the CNS status between both cohorts is shown in Table 1.

**Table 1:** Comparison of CNS status in cohorts A and B *P-value of <0.05 CNS: central nervous system; TLP+: traumatic lumbar puncture

CNS status	Cohort A (2010-2015, n=600)	Cohort B (2015-2018, n=585)	P-value
CNS-1 (n=1,019)	527 (88%)	492 (84%)	0.064
CNS-2 (n=62)	28 (4.6%)	34 (5.8%)	0.376
CNS-3 (n=68)	19 (3%)	49 (8.3%)	0.000*
TLP+ (n=36)	26 (4.3%)	10 (1.7%)	0.009*

The data demonstrate a reduction in the TLP+ cases from 4.3% in cohort A to 1.7% in cohort B (p-value=0.009). However, there was an increase in the incidence of CNS-3 cases in cohort B (8%) as compared to cohort A (3%) (p-value: <0.001). A comparison of CNS status with respect to different risk variables in cohort B is shown in Table 2.

**Table 2:** Comparison of CNS status with respect to risk variables in cohort B CNS: central nervous system; TLP+: traumatic lumbar puncture; WCC: white cell count; ALL: acute lymphoblastic leukemia; NOS: ALL not otherwise specified; NCI: National Cancer Institute; SR: standard risk; HR: high risk

Variables		CNS-1 (n=492)	CNS-2 (n=34)	CNS-3 (n=49)	TLP+ (n=10)	P-value
Age	<10 yrs (n=395)	333 (84%)	25 (6%)	30 (7.5%)	7 (1.7%)	0.687
	≥10 yrs (n=190)	159 (84%)	9 (5%)	19 (10%)	3 (1.6%)	
WCC	<50 x 10^9^/L (n=188)	155 (82%)	12 (6%)	17 (9%)	4 (2%)	0.879
	≥50 x 10^9^/L (n=397)	337 (85%)	22 (5.5%)	32 (8%)	6 (1.5%)	
Phenotype	B-cell ALL (n=467)	399 (85%)	28 (6%)	33 (7%)	7 (1.5%)	0.260
	T-cell ALL (n=115)	91 (79%)	6 (5%)	15 (13%)	3 (2.6%)	
	NOS (n=3)	2 (67%)	0	1 (33%)	0	
NCI risk	SR (n=270)	227 (84%)	20 (7%)	19 (7%)	4 (1.5%)	0.331
	HR (n=315)	265 (84%)	14 (4.5%)	30 (9.5%)	6 (2%)	

The results showed that the incidence of CNS-3 and TLP+ was higher in patients of younger age (less than 10 years), those with a high WCC (more than 50 x 10^9^ white blood cells/L), T-cell ALL phenotype, and a high NCI risk. However, these findings were not statistically significant (p-value: >0.05).

## Discussion

The evaluation of CNS status during chemotherapy (through LP) on Day One and Day Eight, and the administration of intrathecal chemotherapy, is an integral part of ALL treatment protocols. The risk of TLP+ is increased by a number of risk factors at the time of presentation of the disease, such as a high WCC, T-ALL phenotype, younger age, and an unstable clinical condition [6,10,13]. 

Hasegawa et al. adopted a different approach of deferring the initial LP until Day Eight of prednisolone prophase and found a significant decrease in TLP+ as well as CNS-2 and CNS-3 cases. In the present study, we also delayed the first LP till Day Eight of prednisolone prophase. Our study showed a significantly low incidence of TLP+ in cohort B (1.7%) (p-value=0.009), which is comparable with the findings of Hasegawa et al. (0.8%) [12]. The success of this approach is likely due to a decrease in the WCC and the clearance of blast cells from circulation by Day Eight of prednisolone prophase. A comparison of the CNS status of both the cohorts with the internationally published data is shown in Table 3.

**Table 3:** Comparison of CNS status of cohorts A and B with the internationally published data CNS: central nervous system; TLP: traumatic lumbar puncture; LP: lumbar puncture

Study	Year	Number of patients	CNS-1	CNS-2	CNS-3	TLP+
Gajjar et al. [6] (LP on Day One)	2000	546	71%	15%	3%	11%
Bűrger et al. [9] (LP on Day One)	2003	2,012	85%	5%	3%	7%
Hasegawa et al. [12] (LP on Day Eight)	2012	745	90%	2%	2.9%	0.8%
Cohort A (LP on Day One)	2010-2015	600	88%	5%	3%	4%
Cohort B (LP on Day Eight)	2015-2018	585	84%	6%	8%	2%

In contrast with cohort B (0.8%), a significantly higher incidence of TLP+ was found in cohort A (4.3%) (p-value=0.009), which is comparable with the incidence found in the international studies (7-11%) in which LP was performed on Day One [6,9].

In contrast with cohort A and other published studies, we observed a significantly higher incidence of CNS-3 in cohort B (8%) (p-value: <0.005) [6,9,12,14,15]. The cause of this unusual finding can be two-fold: a delay in the diagnosis of CNS-3 cases till Day Eight, and a delay in the first dose of intrathecal chemotherapy that is done along with LP to prevent the contamination of CNS with blast cells. Another explanation can be an inadequate penetration of prednisolone through the CNS. These explanations, however, are not consistent with the findings of the Hasegawa et al. study, which shows only a 1% incidence of CNS-3 cases [12]. Therefore, further prospective studies that compare Day-One with Day-Eight LP are needed.

The present study showed a higher incidence of CNS-3 and TLP+ in patients with high WCC (more than 50 x 10^9^ white blood cells/L), those with T-cell ALL phenotype, and a high NCI risk, which is comparable with the findings of Lee et al. and Smith et al. [16,17]. However, these findings were not statistically significant. The current study also showed a higher incidence of CNS-3 with T-cell ALL phenotype (2.6%) in cohort B as compared with the incidence in international studies, the reason being a high incidence of T-cell ALL in our population, and hence a high proportion of CNS-3 cases [18-20].

There are certain limitations to this study. This was a retrospective, single hospital-based study. The principal limitation of this study was the non-availability of data regarding CNS status with respect to different risk variables (age, WCC, phenotype, NCI risk) in cohort A. Since the incidence of CNS status in cohort A was comparable to that of the international studies whose LP, like in cohort A, was also performed on Day One, we considered cohort A as a historical cohort and assumed its risk factors to be comparable with the internationally published data [6,9]. However, more prospective studies with the inclusion of these risk variables for both cohorts involving multiple institutions need to be conducted in the future.

## Conclusions

TLP+ is a recognized risk factor for the development of iatrogenic CNS leukemia in ALL patients. Its risk increases due to the presence of various factors at the time of presentation of the disease. In the present study, the modified approach of doing the first LP on Day Eight of prednisolone prophase instead on Day One resulted in a significant reduction in TLP+ cases owing to a decrease in WCC and a relatively more stable condition of the patients on Day Eight as compared to Day One. However, the high incidence of CNS-3 in cohort B in this study is an unusual and serious finding, and it warrants more prospective studies before this approach (Day-Eight LP) can be implemented as a standard of care.
